# Safe prone checklist: construction and implementation of a tool for performing the prone maneuver

**DOI:** 10.5935/0103-507X.20170023

**Published:** 2017

**Authors:** Vanessa Martins Oliveira, Daniele Martins Piekala, Gracieli Nadalon Deponti, Danusa Cassiana Rigo Batista, Sílvia Daniela Minossi, Marcele Chisté, Patrícia Maurello Neves Bairros, Wagner da Silva Naue, Dulce Inês Welter, Sílvia Regina Rios Vieira

**Affiliations:** 1 Unidade de Terapia Intensiva, Hospital de Clínicas de Porto Alegre - Porto Alegre (RS), Brasil.; 2 Universidade Federal do Rio Grande do Sul - Porto Alegre (RS), Brasil.

**Keywords:** Respiratory distress syndrome, adult, Prone position/methods, Pronation/methods, Respiratory failure, Check list, Patient safety, Inservice training

## Abstract

**Objective:**

To construct and implement an instrument (checklist) to improve safety when performing the prone maneuver.

**Methods:**

This was an applied, qualitative and descriptive study. The instrument was developed based on a broad review of the literature pertaining to the construction of a care protocol using the main electronic databases (MEDLINE, LILACS and Cochrane).

**Results:**

We describe the construction of a patient safety tool with numerous modifications and adaptations based on the observations of the multidisciplinary team regarding its use in daily practice.

**Conclusion:**

The use of the checklist when performing the prone maneuver increased the safety and reliability of the procedure. The team's understanding of the tool's importance to patient safety and training in its use are necessary for its success.

## Abstract (pt)

**Objetivo:**

Construir e implementar um instrumento (*checklist*) para melhoria do cuidado na manobra prona.

**Métodos:**

Estudo aplicativo, qualitativo e descritivo. O instrumento foi desenvolvido a partir de ampla revisão da literatura, para construção de um protocolo de atendimento assistencial, utilizando as principais bases eletrônicas (MEDLINE, LILACS e Cochrane).

**Resultados:**

Descrevemos a construção de uma ferramenta de segurança do paciente com suas inúmeras modificações e adaptações, a partir das observações da equipe multidisciplinar com seu uso na prática diária.

**Conclusão:**

A aplicação do *checklist* na manobra de prona acrescentou confiabilidade e segurança ao procedimento. O entendimento da importância da ferramenta na segurança do paciente, por parte da equipe, e sua capacitação são necessários para seu sucesso.

## INTRODUCTION

Adult respiratory distress syndrome (ARDS) has high mortality and morbidity, despite technological developments in recent decades. One of the therapies proposed for its treatment is the use of the prone position, which has been studied since 1974 and has gained popularity because it improves hypoxemia in 70% of cases.^(1,2)^ In recent years, interest in the prone position has resurfaced following the publication of a large randomized clinical trial that demonstrated a significant reduction in mortality in the pronated group.^(3,4)^ This finding has significantly increased bedside use of the prone maneuver.

The maneuver is not risk-free. The incidence of complications is small (approximately three per thousand patient/days), but when complications occur they can be fatal, as in cases of central catheter extubation and avulsion. Several complications have been observed, such as pressure ulcers on the face, chest and knee; breast necrosis in patients with silicone prostheses; facial, limb and chest edema; brachial plexus injury; operative wound dehiscence; diet intolerance; accidental extubation; selectivity; endotracheal tube displacement and obstruction; removal of or difficulty of flow in the hemodialysis catheter and other catheters; and the removal of enteral and vesical catheters.^(4,5)^

The most common complications are pressure ulcers, mechanical ventilation-associated pneumonia and endotracheal tube obstruction or decannulation. The most serious fatal event is accidental extubation, which is rare (zero to 2.4% prevalence).^(4-7)^ A recent meta-analysis of the safety and efficacy of the maneuver showed that patients who were pronated had an increased risk of pressure ulcers, endotracheal tube displacement and tracheostomy. However, no significant differences were observed in the occurrence of other complications, such as cardiovascular events or ventilation-associated pneumonia.^(8)^

These results suggest that the procedure is safe and inexpensive but requires teamwork and skill. Thus, centers with less experience may have difficulty managing complications, but nursing care protocols and guidelines can mitigate this risk.^(8)^ Reports in the literature suggest that the incidence of adverse events is significantly reduced in the presence of trained and experienced staff, which makes the maneuver safe.^(9-12)^

An analysis of existing studies reveals some important considerations for clinical practice regarding the need to organize the pronation process. Thus, this study proposes to construct and implement a tool in a checklist format to standardize the process and make the prone procedure safe.^(13)^ Checklists are have been used for many decades in aviation, civil construction and other non-medical areas to guide users when completing tasks in which errors or omissions can be fatal. The application of checklists reduces errors of omission and the improper application of procedures and protocols and creates reliable and reproducible evaluations.^(14-16)^ Similar to flight and military crews, health professionals must often analyze and manage stressful and fatiguing situations.^(17)^ Therefore, in recent years, checklists have also been applied in the health field to improve the quality of medical care.^(18)^ There are several examples of the successful application of checklists in health care areas that require systematic and rapid approaches, such as anesthesiology, surgery, emergency treatment and intensive care.^(18,19)^

The objective of this study was to construct and implement an instrument (checklist) to improve care when performing the prone maneuver.

## METHODS

This was a descriptive, applied, narrative, experience-reporting study that aimed to describe the process developed by the Pronation Teaching and Research Group (*Grupo de Ensino e Pesquisa em Prona* - PEP-PRONA) at a teaching hospital in the city of Porto Alegre, Rio Grande do Sul State (RS), Brazil.

The study was conducted in the intensive care center of the *Hospital de Clínicas de Porto Alegre* starting in the second half of 2015 and was approved by the Ethics Committee (CAAE 61274316.1.0000.5327). The institution's intensive care unit comprises 44 clinical and surgical beds and has a mean hospitalization of 1,800 patients/year. This health organization was chosen mainly due to the presence of a multidisciplinary group composed of physicians, physical therapists, nutritionists and nurses. The group was created in 2012 to implement a protocol for the prone maneuver.

**Figure 1:**
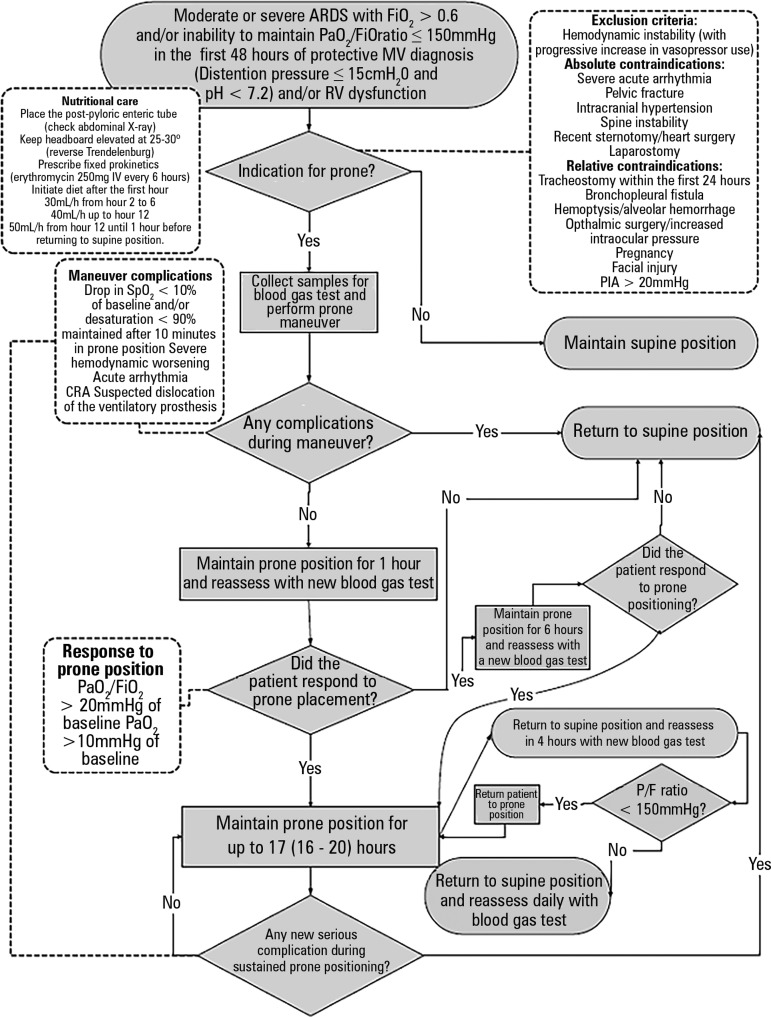
Flow diagram of the prone position care protocol. ARDS - adult respiratory distress syndrome; FiO_2_ - inspired oxygen fraction; PaO_2_ - partial oxygen pressure; MV - mechanical ventilation; IV: intravenous; RV - right ventricle; PIA - intra-abdominal pressure; SpO_2_ - oxygen saturation; CRA - cardiorespiratory arrest; P/F - ratio of partial oxygen pressure to inspired oxygen fraction.

Following a protocol instituted in 2014 that was accompanied by team training with realistic simulation techniques, the need for improvements in the process was identified (Figure 1). The objective was to improve the efficacy of care and patient safety; therefore, the creation of a bedside checklist was proposed. This study describes the standardization of the checklist, its application in the procedure, the difficulties encountered in the process, and the changes made during the tool's construction.

The instrument was based on a care protocol^(20)^ that was constructed on the basis of a broad review of the literature identified with a thorough search of the main electronic databases (MEDLINE, Latin American and Caribbean Health Sciences Literature [Literatura Latino-Americana e do Caribe em Ciências da Saúde - LILACS] and COCHRANE) for the period between January 1995 and March 2016. Original studies or reviews were included, without language restrictions. Studies involving patients under 18 years of age or animals were excluded.^(20)^

The following descriptors were used: (("*prone position*" [MeSH Terms]) *OR Prone* [TextWord]) *OR prone* [Text Word]) *OR proning* [Text Word])) *AND* (("*Intensive Care*" [Mesh]) *OR* "*Intensive Care*" [Text Word]) *AND* ("*Respiratory Distress Syndrome*, Adult" [MeSH Terms]) OR *Respiratory Distress Syndrome, Adult* [Text Word]) *OR* ARDS [Text Word]).^(20)^

The checklist was developed and improved during care for ten patients with moderate and severe ARDS who were subjected to the prone maneuver in the intensive care unit between June 2015 and April 2016. On average, two prone sessions per patient and two supine sessions per patient were performed. The mean time spent in the prone position in each session was 17 hours.

The original instrument required several modifications over time based on the experience gained from the innumerable performances of the maneuver at bedside.

We describe these developments in the organization of the tool and team in table 1.

**Table 1:** Development of the instrument over time

Description	First version	Second version	Third version	Fourth version
Modifications to the instrument suggested by the multidisciplinary team over time	All care was described in sequence without division into pre-, during and post-maneuver activities (standard operating procedure) There were no check boxes for items The tool was not included in the patient folder The instrument was read by a team member involved in the maneuver The team members participating in the process were not specified in advance	Care was separated into pre-, during and post-maneuver periods The checklist’s layout was similar to that of the safe surgery checklist but without boxes to check	Item check boxes were implemented The boxes were still checked by the team members themselves Header with information about the time of pronation and time of return to supine position was added, facilitating the organization of the team It was determined that the instrument should remain in the patient's folder	It was determined that before the application of the tool, team members should determine the times of pronation and return to supine position Written guidelines considered most relevant to the process’s safety were bolded Space for the description of adverse events was added

## RESULTS

Some modifications were proposed in the final version of the instrument with the determination of four steps that should be followed at bedside before starting the checklist.

### Step 1: time and team definition (responsible: physician, nurse and physical therapist)

The physician defines the need for performing the prone maneuver and, together with the nurse and physiotherapist, determines the time of the maneuver and identifies the members of the prone team by name. The team should comprise six members: a physician, a physical therapist, a nurse, two technicians, and a physical therapist or nurse or technician responsible for reading and checking all checklist items. The person responsible for reading the tool should not participate in the procedure. In the case of patients with a chest drain, the team should include one more member, who is responsible for taking care of the drain and bottle.

We recommend that X-rays not be performed in the prone position due to the risk-benefit ratio; namely, the risk of catheter and endotracheal tube avulsion during the examination. Moreover, in this position, interpretation of the results is impaired as most professionals are not accustomed to interpreting images in other positions. Alternatively, thoracic echography can be performed to evaluate the pulmonary parenchyma and catheter position.^(20)^

### Step 2: provide cushions (responsible: physical therapist)

**Figure 2:**
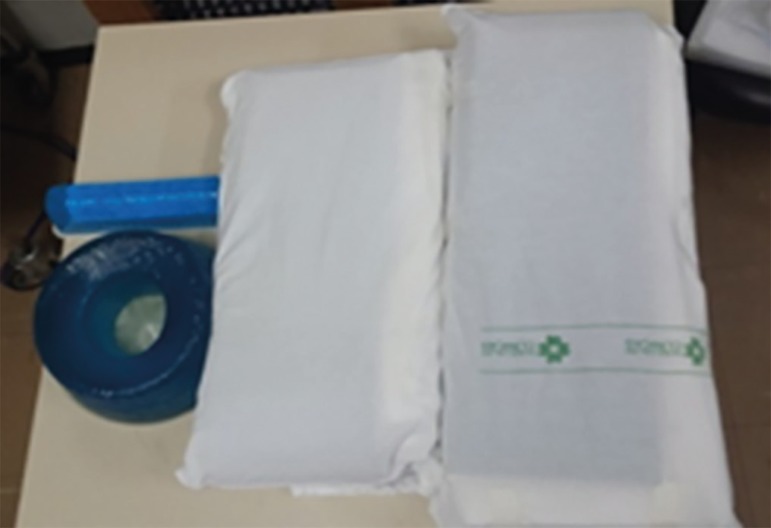
Cushions for face, chest, pelvis and wrist.

Once the need for the maneuver has been identified, the physiotherapist prepares or provides cushions to support the face, chest, pelvis, wrists and anterior leg region (Figure 2).

### Step 3: pre-maneuver care (responsible: nurse)

The nurse performs the time-in (pre-maneuver care) steps, which are checked when the whole team is assembled.

### Step 4: team assembles to perform the maneuver

At the time predetermined by the team, all the designated professionals must assemble. The physician be positioned at the head of the bed to coordinate the rotation and to promptly reintubate the patient in case of accidental extubation. The nurse and physical therapist should stand on each side of the patient's trunk. Two technicians should position themselves on either side of the patient, next to the legs.^(20)^ In the case of an obese patient, two more people can be added to the team. A team member who is not involved in the maneuver should perform the checklist.

After these four steps are completed, the safe prone checklist is started. The checklist is divided into pre-maneuver care (time in)*,* performance of the maneuver and post-maneuver care (time out).

### Pre-maneuver care

The nurse and the technician perform some tasks before the designated time for commencing the maneuver. These tasks should be checked again at checklist time. The tasks are divided into nutritional care (suspend feeding and open the nasoenteric tube 2 hours before the procedure); material care (provide cushions; place the crash cart and intubation unit close by; test aspiration equipment and bag-valve-mask device [AMBU]); general care (provide eye and skin care, review the fixation of invasive and curative devices, suspend continuous hemodialysis [recirculate and heparinize catheter]); airway care (airway aspiration; check fixation of the cord; record mouth corners and cuff pressure of the endotracheal tube; pre-oxygenate the patient with inspired oxygen fraction - FiO_2_: 100% for 10 minutes); and analgesia and sedation care (assess the need for increased sedation and curarization [evaluate the bispectral (BIS) index value, when available]).^(5,20)^

In the first version of the tool, the nurse's and physiotherapist's actions at the beginning of the maneuver, when the team positions itself and the checklist is performed again, were not determined, nor was the care performed prior to the beginning of the maneuver. However, separating the tasks and taking these precautions before beginning the maneuver expedites the procedure time. In the initial tool, the items were verbally checked but not confirmed with the team as a whole or annotated. The instrument was read by a team member involved in the maneuver. By checking at the time of the maneuver, when all the professionals are in position, and having another professional read the checklist aloud and marks each checked item, we observed a gain in time and organization and found that more attention was paid to the process.

### Care in the performance of the maneuver

**Figure 3:**
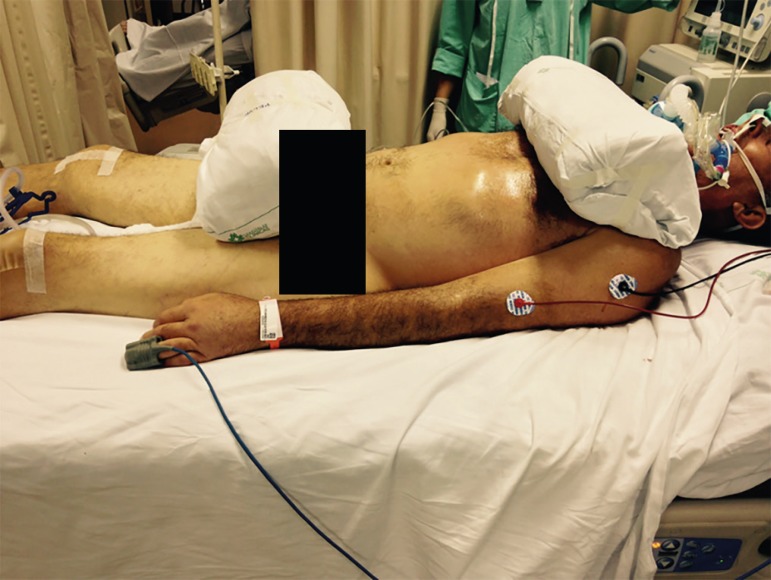
Placement of the cushions on the chest and pelvis before the envelope maneuver is performed.

**Figure 4:**
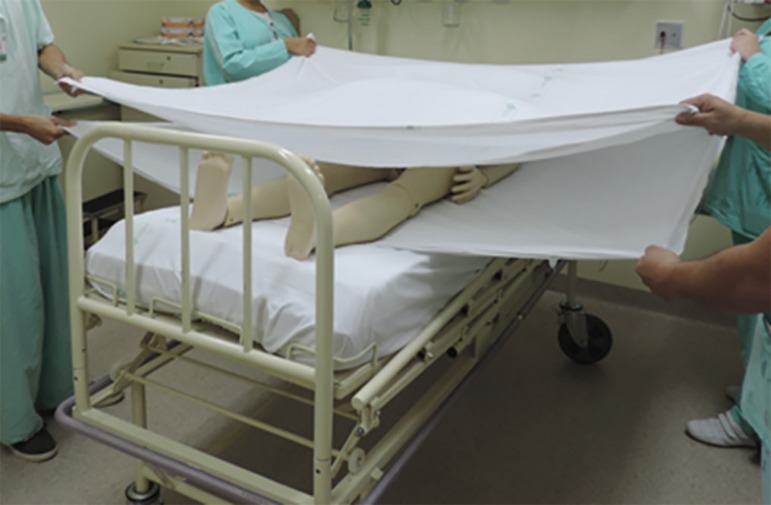
Envelope Maneuver. Step 1: Position the top sheet over the lower sheet. Place drains, tubes and invasive pressure transducer inside the envelope.

**Figure 5:**
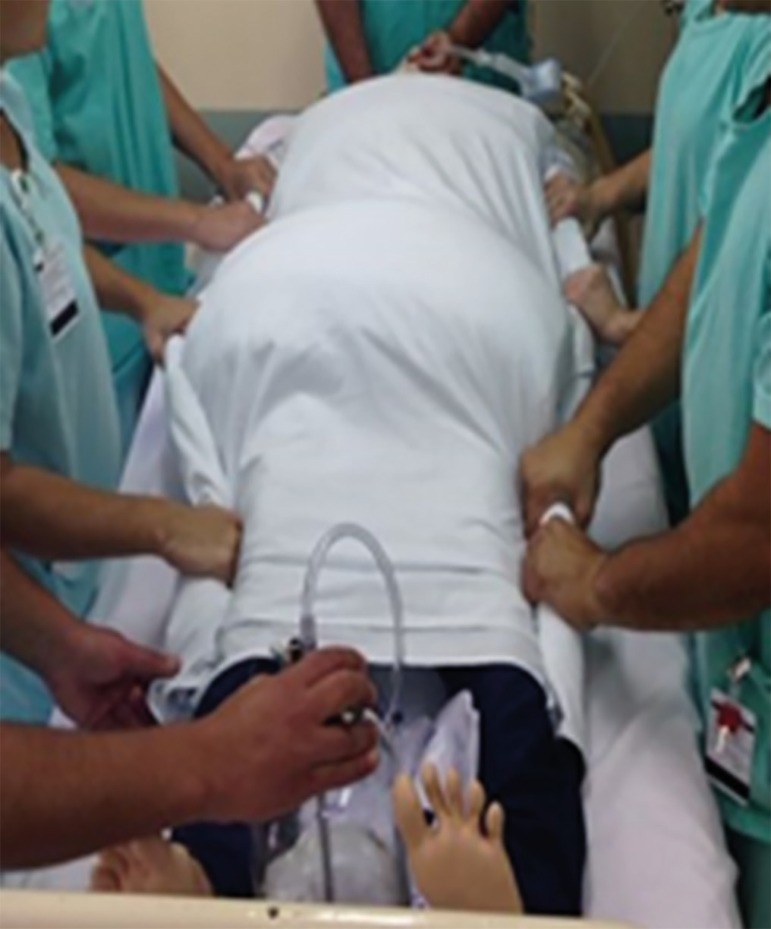
Envelope Maneuver. Step 2: Join and wrap the top and bottom sheet as closely as possible to the patient's body.

Before the maneuver is performed, the second part of the checklist is applied (confirmation). It is confirmed that the entire team is in the correct position (physician at the headboard and the other group members distributed along the two sides of the bed) and that everyone knows the envelope maneuver and the three turning points. The tool is then read, and the signal readings for the maneuver (place invasive blood pressure electrodes and transducer on the upper limbs and align monitoring and oximetry cables; disconnect BIS ventilator if in use; disconnect the nasoenteric tube from the bottle and close; disconnect the aspirator; clamp tubes and drains and place them between the patient's legs or arms) are checked. Next, the performance procedures are read aloud (place head in a flat position and align limbs, position the pelvis and chest cushions, and suspend and disconnect infusions), and the envelope is formed (Figures 3 to 5).^(4,5,20)^

**Figure 6:**
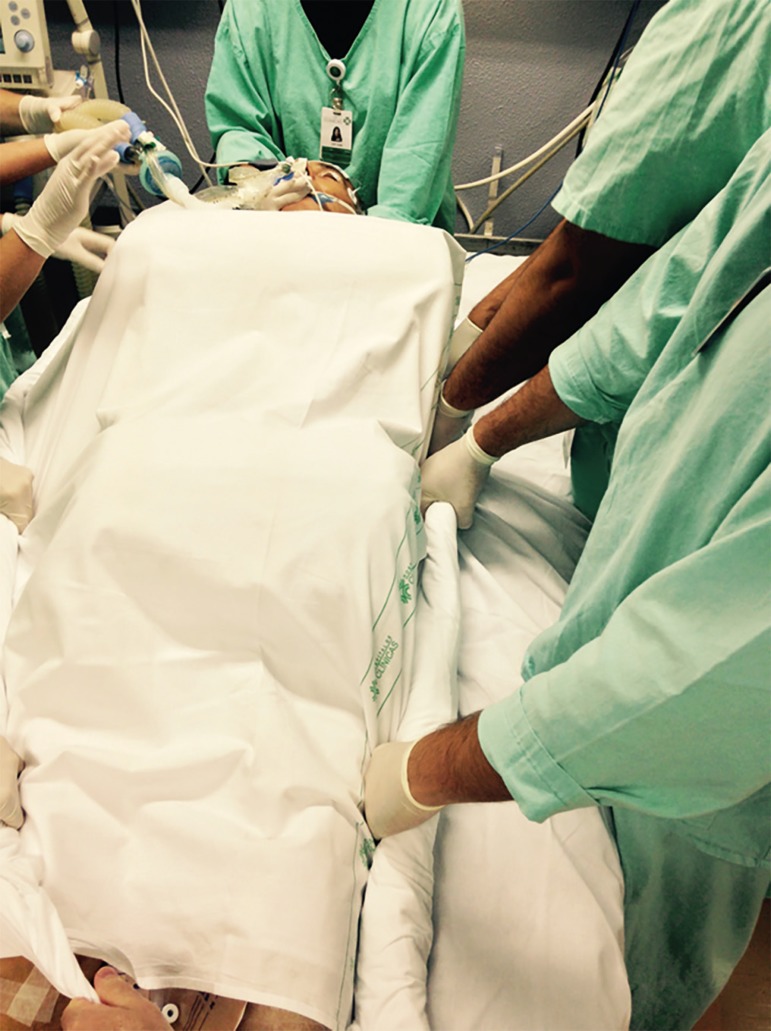
Envelope Maneuver. Step 3: Start turning the patient on the physician's command. Move the patient to the side of the bed opposite the mechanical ventilator.

**Figure 7:**
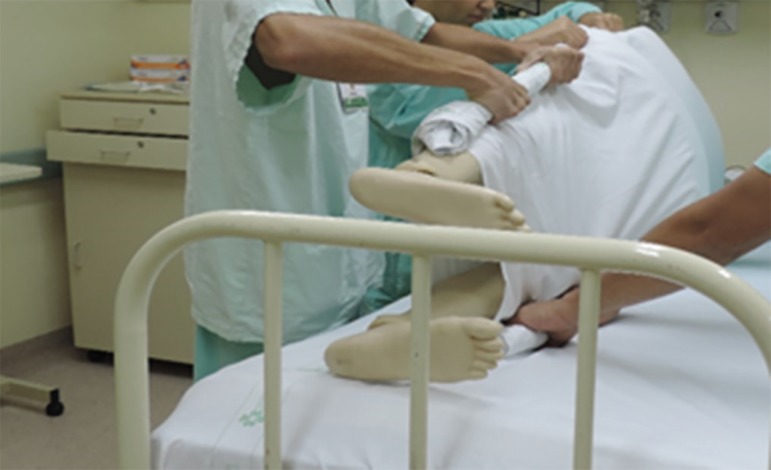
Envelope Maneuver. Step 4: Turn the patient to lateral position. Perform the hand exchange maneuver among the team by placing one hand on the left side and one on the right side of the patient.

**Figure 8:**
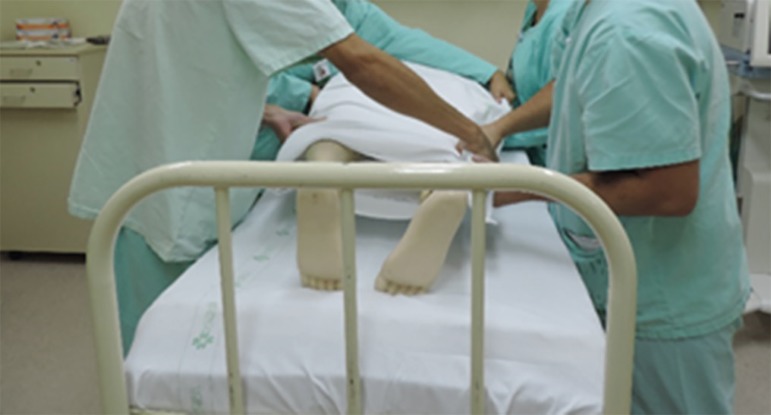
Envelope Maneuver. Step 5: End of rotation and prone positioning and start of post-maneuver care.

The three-point turn is performed on the physician's command.^(20)^ The patient must be moved to the side of the bed opposite the mechanical ventilator, placed in lateral position, and then turned to the prone position. (Figures 4 to 8).

**Figure 9:**
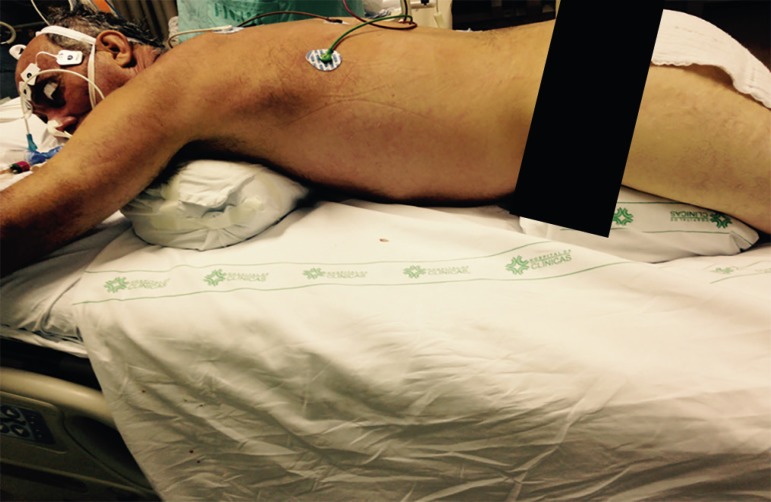
Post-maneuver care (check the placement of the cushions, keeping the abdomen free).

The checklist also covers the reporting of adverse events before, during, and after the procedure (Figures 5 to 9). No adverse events were observed in this group of patients.

### Post-maneuver care

After the procedure, with the patient already in the prone position, the positioning of the endotracheal tube by pulmonary auscultation and mouth corners is checked. The tube cuff pressure is confirmed. It is also necessary to check the position of the pelvis and anterior chest cushions, ensuring that the abdomen is free, and to check the positioning of the other cushions: face (avoiding eye and ear injuries and breakage of the endotracheal tube), hand, and anterior leg region (Figure 9).^(4,6,20)^

**Figure 10:**
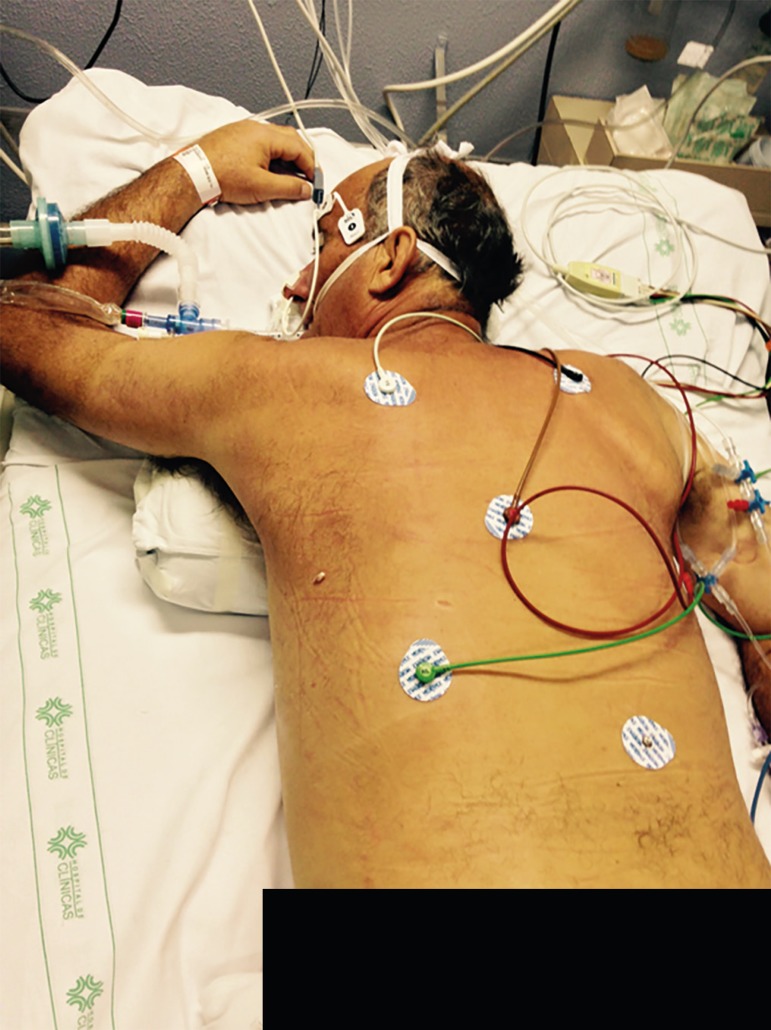
Swimmer's position (one arm raised and head rotated toward the raised arm; the other arm is positioned alongside the body).

The position of the headboard (reverse Trendelenburg) is checked to reduce the risk of aspiration. The invasive arterial pressure transducer and electrodes on the patient's chest must be repositioned. The upper limb is raised into the swimmer's position and alternated every 2 hours to avoid injury to the brachial plexus (Figure 10).^(4-6,20)^

**Figure 11:**
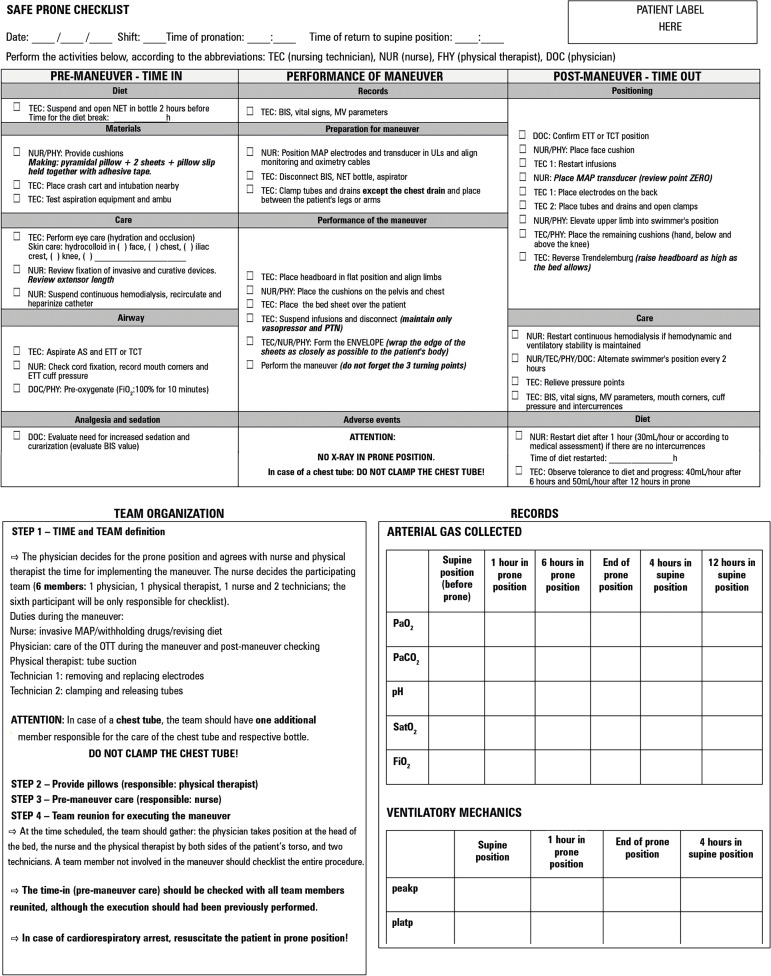
Latest version of the safe prone maneuver checklist (time in, performance of maneuver and time out). Front and back of the sheet with guidelines for the team and prone protocol in flowchart format. NET - nasoenteric tube; BIS - bispectral index; MV - mechanical ventilation; ETT - endotracheal tube; TCT - tracheostomy; FiO_2_ - inspired fraction of oxygen; MAP - invasive mean arterial pressure; ULs- upper limbs; PTN - parenteral nutrition; AS - airways; PaO_2_ - partial oxygen pressure; PaCO_2_ - partial carbon dioxide pressure; pH - hydrogen ion concentration; SatO_2_ - oxygen saturation; peakp - peak pressure; platp - plateau pressure; PEEP - positive end-expiratory pressure.

Parenteral infusion and hemodialysis drugs, if used, are restarted. Pressure points are relieved, especially in the iliac crests and knees. Vital signs are again recorded, and the re-initiation of enteral feeding is re-evaluated during the second hour in prone position if there are no complications (Figure 11).^(20)^

**Figure 12:**
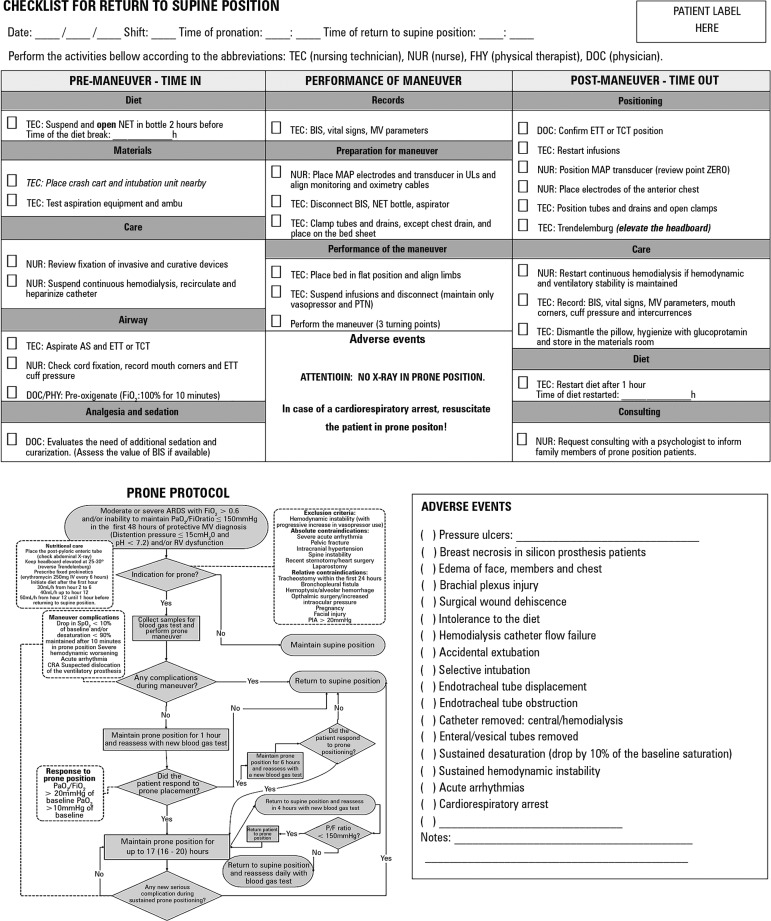
Front and back of the checklist for return to supine position. NET - nasoenteric tube; BIS - bispectral index; MV - mechanical ventilation; ETT - endotracheal tube; TCT - tracheostomy; MAP - invasive mean arterial pressure; ULs - upper limbs; PTN - parenteral nutrition; AS - airways; FiO_2_ - inspired oxygen fraction; ARDS - adult respiratory distress syndrome; RV - right ventricle; SpO_2_ - oxygen saturation; PaO_2_ - partial oxygen pressure; PIA - intra-abdominal pressure; P/F - ratio of partial oxygen pressure to inspired oxygen fraction.

During the return-to-supine position maneuver using the safe prone checklist, we observed a number of obsolete items that made the instrument lengthy and confusing. Therefore, to facilitate the process, a checklist was proposed for returning the patient to the supine position (Figure 12).

We observed that the team had great difficulty agreeing on a time to return the patient to the supine position. Therefore, we included the time that prone began and the time at which the patient should be supinated on the form header. This decision should be made by the team when it is together (preferably during the day) considering a range of 17 - 20 hours in prone position, as suggested by the literature.^(3)^ The date and time of pronation and date and time of return to the supine position should be recorded on the header of both the safe prone and supine position checklists.

To apply the latest version of the checklist, the team was previously trained using realistic simulation techniques and a focus group to develop technical skills and team control in emergency situations.

## DISCUSSION

Checklists are among the many tools used in practice to support the multidisciplinary team. Checklist use increases process safety by organizing the basic criteria to follow and condensing a large amount of knowledge into a concise format.^(21,22)^ Essential criteria that the user of a particular process must remember should be included in the tool to increase the objectivity of the process's evaluation and reproducibility.^(23,24)^

This tool is a perfect fit for the prone maneuver as this procedure is not frequently applied in daily practice and requires numerous precautions that, if forgotten or performed poorly, can endanger the patient.

However, the excessive use of checklists can become an obstacle rather than a support resource and error management tool. Professionals may experience "checklist fatigue" when checklists are used unnecessarily or are excessively lengthy. Therefore, the careful selection of checklist topics and consideration of clinical judgment in content construction are necessary.^(22,23)^ It is important to consider that checklists are not appropriate in all environments and should be used for tasks that are prone to error or omission to improve accuracy, adherence to best practices and the reliability of the process.

The list should be easy and practical, giving health professionals the freedom to use their clinical judgment. It should not interfere with patient care time. The checklist should be reviewed frequently to ensure that it reflects the difficulties that the team encounters in practice and to perform updates based on current evidence from the literature.^(22,23)^

More than a list, the checklist is a tool that should be built by the team, and only items that add value to the process should be included. A major challenge at bedside is overcoming the stigma that checklists are an imposition; the group should be shown that the use of the checklist contributes to patient safety. The fact that the team is aware of the checklist does not mean that it knows how to use it. The reason the checklist should be used and how to use it properly should be shown to all team members through training.^(22,23)^ Repeated application of this tool is important to identify team difficulties and to suggest improvements to the instrument.

## CONCLUSION

The application of the checklist when performing the prone maneuver made the maneuver safer and more reliable. It is necessary to involve the entire team in the check so that everyone respects each of the items on the list and is aware that performing them is essential to the success of the maneuver. Communication is central to success, and the checklist makes this happen in the best possible way.

The frequency of the tool's use and its adaptation to the reality of each unit where it is implemented should be taken into account.
